# Burkitt’s lymphoma masquerading as appendicitis – two case reports and review of the literature

**DOI:** 10.1186/1477-7819-12-187

**Published:** 2014-06-18

**Authors:** Elroy P Weledji, Marcelin N Ngowe, John S Abba

**Affiliations:** 1 Department of Surgery, Faculty of Health Sciences, University of Buea, P.O. Box 12, Buea, S.W. Region, Cameroon; 2 Tiko Cottage Hospitals, Cameroon Development Corporation, Tiko, S.W. Region, Cameroon

**Keywords:** Appendicitis, Burkitt’s lymphoma, Histology, Treatment

## Abstract

Two cases of Burkitt’s lymphoma masquerading as appendicitis are reported herein. The diagnoses were made post-operatively from the appendix specimen in one case and from an ileocecal resection specimen for cecal fistula complicating an appendicectomy in the second case. These cases highlight the importance of routine histological examination of appendicectomy specimens.

## Background

Burkitt’s lymphoma is an unusual variant (5%) of malignant (B-cell) non-Hodgkin’s lymphoma, which was first described between 1958 and 1962 in children in sub-Saharan Africa [1]. Over half of these patients presented with jaw tumours, but it was soon realised that other organs, including distal ileum, caecum, ovaries, kidney, or the breast, were often involved and that the initial presentations were variable. Non-Hodgkin’s lymphoma is the most common malignant tumour of the bowel in children older than 5 years but Burkitt’s lymphoma presenting as appendicitis is rare [2]. Untreated cases run a rapid downhill course and die with wide-spread metastases to the liver, kidneys, and other organs [3].

The epidemiological and laboratory findings suggest that that the combination of recurrent *P. falciparum* malaria and Epstein-Barr virus (EBV) infection very early in childhood cause B cell hyperplasia which is an essential component of lymphomagenesis [4,5]. A sporadic form of Burkitt’s lymphoma occurs elsewhere in the world and has only a 5 to 15% degree of association with EBV. A third form, human immunodeficiency virus (HIV)-associated Burkitt’s lymphoma is associated with EBV in approximately 40% of cases [4,6]. If suspicious of intra-abdominal Burkitt’s lymphoma especially in an endemic area, pre-operative imaging with ultrasonography in expert hands or computed tomography (CT) is effective at diagnosing the malignant nature of the tumour [7,8]. Histological examination of the appendix specimen following appendicectomy for an apparent appendicitis is mandatory as it may disclose an ileocecal Burkitt’s lymphoma.

## Case presentation

### Case 1 presentation

**Figure 1:**
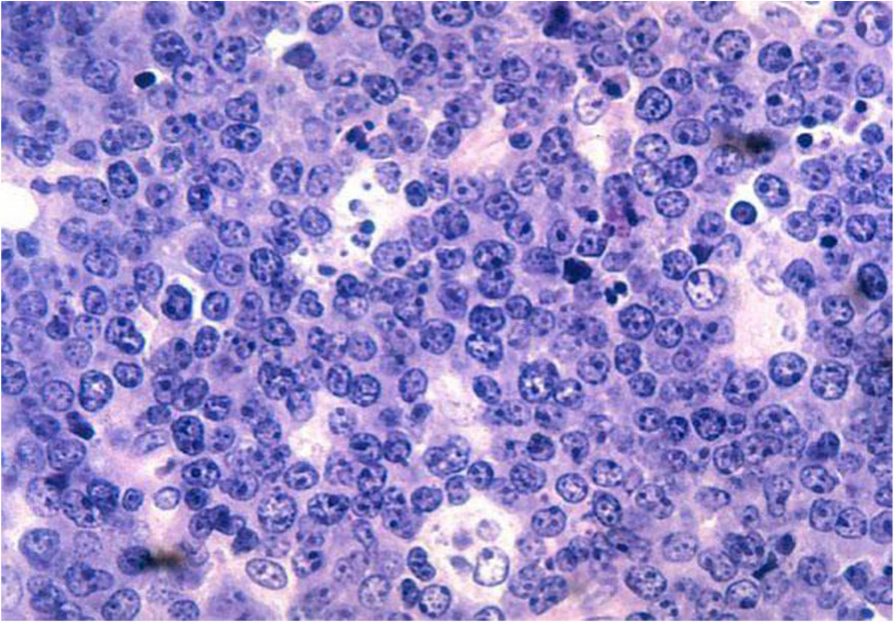
**The Starry-sky pattern of Burkitt’s lymphoma.** The tumour cells of B lymphoblasts which are large rounded or indented nucleus and 3 or 4 nucleoli with thin layer of basophilic cytoplasm are closely apposed to each other forming a dark blue background (the ‘sky’). The surrounding macrophages with abundant pale foamy cytoplasm scattered among the tumour cells (the stars).

A 13-year-old female was admitted electively for investigation of a long history (3 years) of right iliac fossa pain. This was a recurring dull and aching pain that had not responded to analgesia and antibiotics for suspected relapsing or ‘chronic’ appendicitis. She had no anorexia or weight loss. She had no constitutional symptoms, such as lethargy, fever, or night sweats, and had no altered bowel habits or urinary symptoms. On examination she appeared clinically well. The abdomen was soft but tender on deep palpation of the right iliac fossa. Thefull blood count and urinalysis were normal. Ultrasound examination suggested an appendicitis. At operation, a Lanz incision was made in the right iliac fossa. There was some free fluid in the peritoneal cavity but the appendix did not appear inflamed. It simply had a yellowish tinge. There was terminal ileal lymphadenopathy. The histology of the appendix specimen revealed a B-cell non-Hodgkin’s lymphoma consistent with Burkitt’s lymphoma (Figure 1). She made full postoperative recovery and underwent a 6-month course of chemotherapy (i/v cyclophosphamide and vincristine together with oral prednisolone) having tested as HIV seronegative prior to its commencement. Eight years later, she is very well and has graduated as an accountant.

### Case 2 presentation

An 18-year-old female was referred as an emergency with a cecal fistula complicating an appendicectomy performedtwo weeks previously. The indication for the appendicectomy was for an apparent appendicitis following a vague history of right iliac fossa pain. An attempted closure of the fistula failed. On examination, she was clinically dehydrated and exhausted but not toxic. She had no constitutional symptoms and the vital signs were normal. There was a persistent and high output (greater than 1 L/24 h) intestinal fistula in the right iliac fossa consistent with a cecal fistula. She was resuscitated with appropriate fluids and because of the persistent output from the high-pressure caecum a decision was made for surgical correction two days later. At laparotomy, there was a cecal fistula from the dehisced appendix stump. Apart for significant ileocecal lymphadenopathy the rest of the abdomen appeared normal. Following extensive lavage of the operation field with saline solution, an ileocecal resection with end-to-end anastomosis of the ileum and ascending colon was performed. The histology of the operative specimen confirmed a B-cell non-Hodgkin’s lymphoma consistent with Burkitt’s lymphoma. She had a stormy post-operative recovery and when fully recovered underwent a 6-month course of chemotherapy (i/v cyclophosphamide and vincristine together with oral prednisolone) having tested as HIV seronegative prior to its commencement. Nine years later she is well with no evidence of metastases but with fertility problems.

## Discussion

Burkitt’s lymphoma is a highly aggressive B-cell non-Hodgkin’s lymphoma and is the fastest growing human tumour [9,10]. This EBV-associated lymphoma was one of the first tumours shown to have a chromosomal translocation (*chromosome 14*) that activates an oncogene (c-MYC) [4]. The incidence is very high with an aggressive clinical course in immunosuppressed patients in non-endemic areas especially when associated with HIV infection. They were all positive for EBV in a case series [11]. Clinically, Burkitt’s lymphoma patients do not usually exhibit the B-symptoms (fever, night sweats, and weight loss) characteristic of early non-Hodgkin’s lymphoma. This is because the transformation of these cells compromises host defence and evolves mechanisms to escape immune surveillance [12]. Patients with a rapidly growing extranodal intra-abdominal Burkitt's tumour present with symptoms of bowel obstruction, intussusception, or appendicitis [13]. Both cases reported here gave an atypical history for appendicitis especially regarding the duration of the abdominal pain [14]. The differential diagnosis would include a true relapsing or chronic appendicitis which are rare and often difficult to diagnose as the symptoms may be atypical and short-lived [15]. Because of resource limitations, the diagnoses of Burkitt’s lymphoma in these cases were post-operative. In the first case the diagnosis was made from the histological examination of the appendix specimen. In the second case, where an ileocecal Burkitt’s lymphoma masqueraded as an appendicitis, the diagnosis was made only following the histological examination of the resected ileocecal specimen. The demonstration of a malignant appendix by preoperative imaging with ultrasonography or CT would influence the surgical approach for pathological staging of the disease [16]. A bone marrow biopsy may show infiltration by Burkitt’s lymphoma (dissemination) [17]. The diagnosis may also be established by laparoscopy following which the appendix can be removed [18]. Due to Burkitt’s lymphoma’s rapid doubling time, aggressive chemotherapy is required to control its spread and growth [19,20]. Paradoxically it is the high grade non-Hodgkin’s lymphoma that may be curable and consequently should be treated with high-dose multidrug regimens in addition to malaria prophylaxis in Burkitt’s lymphoma [20]. Intensive chemotherapy with CODOX-M/IVAC (cyclophosphamide, vincristine, doxorubicin, and high dose methotrexate, alternating with ifosfamide, etoposide, and cytarabine) is the definitive treatment [17,19,20]. The improved chemotherapy and support care available are due to the better understanding of the biology of the disease [12,17]. The outcome has improved and is now excellent in children; the current 5-year survival for advanced (disseminated) Burkitt’s lymphoma in children and adolescents has increased by 2- to3-fold in the past 3 decades to 85% to 90% following less than 6 months of intensive chemotherapy [9,10].

The two patients in the case report were tested as being HIV seronegative prior to commencing the above standard chemotherapy regimen. The successful outcome in these patients may perhaps not have been achieved if they were HIV seropositive in terms of disease progression and response to chemotherapy [11,21]. Adjuvant CD20-directed monoclonal antibody therapy with rituximab shows promise for improving outcome and reducing the toxic effects of cytotoxic chemotherapy. Rituximab decreased the recurrence rate and showed a trend in favour of improvement in overall survival or disease-free survival [22]. CODOX-M/IVAC with rituximab is a highly effective regime for the treatment of adult Burkitt’s lymphoma which usually has a poor prognosis [23]. Current risk factors in the prognosis of children and adolescent Burkitt’s are elevated lactate dehydrogenase greater than twice the upper limit, bone marrow and central nervous system involvement, poor response to cytoreduction therapy, and poor risk cytogenetics [3,9,10]. Radiotherapy is used only in emergencies such as superior vena cava syndrome, acute neurological impairment, or in patients with relapse [1,3,9,10]. Further investigations including CT and PET scanning for response evaluation in these patients would be possible in a resourced area. There is a need for more clinical data on the use of PET and magnetic resonance technology in the determination of response evaluation of children with Burkitt’s lymphoma [3,16].

## Conclusions

Although Burkitt’s lymphoma presenting as appendicitis is rare, it should be considered an important differential diagnosis in children and adolescents from sub-Saharan Africa with atypical right iliac fossa pain. The reported cases demonstrate the importance of histologically examining appendicectomy specimens especially in patients with atypical presentations of appendicitis. The appendix, being a lymphoid organ, may be the most appropriate and accessible extranodal tissue for diagnosing intra-abdominal Burkitt’s lymphoma or other diseases. Understanding the underlying biology of Burkitt’s lymphoma would lead to strategies for prevention and approaches to reduce acute and chronic toxicities from current cytotoxic treatment.

## Consent

A written informed consent was obtained from the patient for publication of this case report and any accompanying images. A copy of the written consent is available for review by the editor-in-chief of the journal.

## Consent (Child)

A written informed consent was obtained from the patient’s parent for the publication of this report and any accompanying images.

## Abbreviations

CT: Computed tomography; EBV: Epstein-Barr virus; HIV: Human immunodeficiency virus; PET: Posittron emission tomography; MRI: Magnetic resonance imaging.

## Competing interests

The authors declare that they have no competing interest.

## Authors’ contribution

EPW is the main author and drafted the manuscript. MNN gave advice on the tropical perspective and helped with the literature search. JSA helped with the literature search. All authors read and approved the final manuscript.
